# The Role of Carbon Nanoparticles in Lymph Node Dissection and Parathyroid Gland Preservation during Surgery for Thyroid Cancer: A Systematic Review and Meta-Analysis

**DOI:** 10.3390/cancers14164016

**Published:** 2022-08-19

**Authors:** Georgios Koimtzis, Leandros Stefanopoulos, Vyron Alexandrou, Nikos Tteralli, Verity Brooker, Awad Ali Alawad, Eliot Carrington-Windo, Nikolaos Karakasis, Georgios Geropoulos, Theodosios Papavramidis

**Affiliations:** 1 Cardiff Transplant Unit, University Hospital of Wales, Cardiff and Vale University Health Board, Cardiff CF14 4XW, UK; 2 Lab of Computing, Medical Informatics and Biomedical Imaging Technologies, Aristotle University of Thessaloniki, 54124 Thessaloniki, Greece; 3 Department of Electrical and Computer Engineering, Technological Institute, Robert R. McCormick School of Engineering and Applied Science, Northwestern University, 2145 Sheridan Road, Evanston, IL 60208, USA; 4 Urology Department, General Hospital of Thessaloniki “G. Gennimata-Agios Dimitrios”, Elenis Zografou 2, 54634 Thessaloniki, Greece; 5 Department of General Surgery, North Hampshire NHS Foundation Trust, Basingstoke RG24 9NA, UK; 6 Emergency Department, Grafton Base Hospital, Northern NSW Local Health District, Grafton, NSW 2460, Australia; 7 Department of Academic Surgery, The Royal Marsden Hospital Foundation Trust, 203 Fulham Rd., London SW3 6JJ, UK; 8 1st Propedeutic Surgical Department, University Hospital of Thessaloniki AHEPA, Aristotle University of Thessaloniki (AUTH), 1st St. Kiriakidi Street, 54621 Thessaloniki, Greece

**Keywords:** thyroid cancer, carbon nanoparticles, lymph nodes, parathyroid glands

## Abstract

**Simple Summary:**

The incidence of thyroid cancer has been increasing over the past few years. Surgery remains still the primary therapeutic option and lymph node dissection is often required. The aim of this study was to perform a meta-analysis of the available data on carbon nanoparticles, a novel agent that can be used to identify lymph nodes intraoperatively more efficiently, while also promoting the protection of the parathyroid glands by reducing the rate of accidental removal. Based on our study, carbon nanoparticles significantly increase the number of lymph nodes harvested during thyroid surgery, while they decrease the accidental removal of parathyroid glands removal rate by 34%. Thus, we suggest their use on a wider basis so that more data can be available for future research on the subject.

**Abstract:**

Thyroid cancer is the most common endocrine malignancy with an increasing incidence over the past few years. Surgery is considered the primary therapeutic option, which often involves lymph node dissection. The aim of this study was to assess the role of carbon nanoparticles, a novel agent, in thyroid cancer surgery. For that purpose, we conducted a systematic review of the literature on MEDLINE, EMBASE, Scopus, Cochrane and Google Scholar databases from 1 January 2002 to 31 January 2022. Ultimately, 20 articles with a total number of 2920 patients were included in the analysis. The outcome of the analysis showed that the use of carbon nanoparticles is associated with a higher number of harvested lymph nodes (WMD, 1.47, 95% CI, 1.13 to 1.82, *p* < 0.001) and a lower rate of accidental parathyroid gland removal (OR 0.34, CI 95% 0.24 to 0.50, *p* < 0.001). Based on these results, we suggest that carbon nanoparticles are applied in thyroid cancer surgery on a wider scale, so that these findings can be confirmed by future research on the subject.

## 1. Introduction

Thyroid cancer is the most common endocrine cancer [1] and its incidence has increased significantly during the last two decades [2]. Thyroid cancer is the fifth most common cancer in the female population with a 1:3 male-to-female ratio [3]. In general, thyroid malignancies affect the younger population with a median age of 51 years at the time of diagnosis, while 43% of new cases are patients between 45 and 64 years. While the increase in the incidence of thyroid cancer is estimated to be 300% globally, mortality rates have decreased or remained stable [3,4]. The overall 5-year survival rate is 98.1% and reaches 99.9% for localized cases [3]. Nonetheless, for distant disease it drops to 55.5% [3].

Thyroid malignancies originate either from the follicular epithelial cells or from the parafollicular C cells [1]. Follicular derived thyroid cancers are mainly divided into differentiated cancers, which account for 95% of the cases, and anaplastic thyroid cancers, which are extremely rare (<1%) [5]. The most common subtype of well-differentiated thyroid cancer is papillary thyroid cancer, and follicular thyroid cancer and Hurthle cell thyroid cancer also fall in this category [5]. The majority of papillary thyroid carcinomas have an excellent prognosis, although high recurrence rates of 25–35% have been reported [6]. Papillary thyroid cancer usually metastasizes in the cervical lymph nodes [5] and lymph node metastatic disease has been proven to be a considerable risk factor in developing recurrent disease, rendering lymph node positivity an important factor in planning the appropriate treatment strategy [7]. According to the guidelines of the European Society of Medical Oncology (ESMO), prophylactic central neck dissection may improve regional control in cases of more invasive tumors [8], and the same recommendation comes from the American Thyroid Association [9]. Moreover, lateral neck dissections (radical or modified) are recommended as necessary therapy for patients with radiologically or histologically proven metastatic lymph node disease in the lateral cervical compartments [9,10]. It is therefore evident that the intraoperative identification and resection of metastatic lymph nodes is crucial to achieve the optimal therapeutic outcome for the patient.

The galloping advances in the field of nanotechnology have been continuously providing more and more nanomaterials for use in the field of cancer diagnosis and management [11]. Carbon nanoparticles play a significant role, since they can serve as carriers of various types of drugs, be used in the reconstruction of bone tissue, contribute to the early diagnosis of cancer cells, have anti-inflammatory and antibacterial properties, act as markers in imaging diagnostics and facilitate tracing of lymph nodes in various operations [11,12]. So far, carbon nanoparticles have been successfully used for lymph node tracing in endometrial cancer, gastric cancer, colorectal cancer, breast cancer and thyroid cancer [13,14,15,16,17]. The administration of carbon nanoparticles can also be combined with indocyanine green, which further increases the efficiency of the technique [17,18].

More specifically, in cases of thyroid cancer, the administration of carbon nanoparticles has another significant advantage. Carbon nanoparticle suspension is composed of nanosized polymeric carbon granules that have an average diameter of 150 nm [17]. Because of their size, carbon nanoparticles can pass through the lymphatic vessels that have a diameter of 120–500 nm and be absorbed by macrophages accumulating in lymph nodes, but cannot enter blood capillaries that have a diameter of 20–50 nm [17,18]. As a result, lymph nodes are stained black and are more easily identified and resected, but the parathyroid glands that have a different lymphatic drainage from the thyroid gland are not stained [19]. Therefore, carbon nanoparticles can also be used to identify and protect parathyroid glands during thyroid surgery [19].

The technique for the utilization of carbon nanoparticles in thyroid surgery has been well described in the literature. Following dissection of the strap muscles and exposure of the lesion site of the thyroid gland, a 1 mL syringe is usually used to inject the carbon nanoparticle suspension into the thyroid [18,19]. The lateral and posterior sides of the thyroid gland are not routinely dissected, in order to avoid damage to the lymphatic drainage of the gland [18]. The suspension can either be injected through a single or through multiple points. The injection spot can either be cauterized and sealed by using an electric coagulation scalpel immediately after the syringe is removed from the gland, or by applying appropriate manual pressure in order to prevent solution leakage to the surrounding operating field [18,19]. The surgical dissection usually is carried out five minutes later as the thyroid gland is stained black, followed by the lymph nodes. Nonetheless, in cases of endoscopic approach, the injection of the carbon nanoparticles suspension is usually performed the day before the operation under ultrasound guidance [20].

Despite the abundance of articles in the literature regarding the use of carbon nanoparticles in thyroid surgery, there are still controversial matters, especially among experienced surgeons in high volume centers [19]. There have been reports that carbon nanoparticles do not offer any significant advantage in parathyroid gland protection, while leading to a significantly prolonged operative time [21]. Moreover, other articles report that the use of carbon nanoparticles does not improve the long-term outcomes after thyroid cancer surgery, such as the rates of permanent hypoparathyroidism and recurrence of papillary thyroid carcinoma [22]. The latest systematic review performed on the subject concludes that the use of carbon nanoparticle suspension may lead to a more extensive lymph node dissection, while it improves preservation of the parathyroid glands [23]. Nonetheless, more recent randomized controlled trials have now been published and as a consequence, another systematic review and meta-analysis is required in order to determine if the use of carbon nanoparticles is of any benefit in thyroid cancer surgery.

## 2. Materials and Methods

This study is a systematic review and meta-analysis that was performed without a registered pre-existing protocol. A thorough and detailed online search of the literature was performed to identify articles on the performance of carbon nanoparticles infusion in lymph node dissection and parathyroid gland preservation during surgery for thyroid malignancy. The MEDLINE, EMBASE, Scopus, Cochrane and Google Scholar databases were searched for relevant articles from 1 January 2002 to 31 January 2022. Earlier studies were considered outdated and superseded by more recent research and therefore were not included in the original search. Also, additional search for gray literature was performed on the websites of international surgical, endocrine and oncological associations and networks and on available data from relevant conferences. The following search string was used to search the online databases:

(“carbon” [MeSH Terms] OR “carbon” [All Fields] OR “carbons” [All Fields] OR “carbon s” [All Fields] OR “carbonates” [MeSH Terms] OR “carbonates” [All Fields] OR “carbonate” [All Fields] OR “carbonated” [All Fields] OR “carbonating” [All Fields] OR “carbonation” [All Fields] OR “carboneous” [All Fields] OR “carbonization” [All Fields] OR “carbonizations” [All Fields] OR “carbonize” [All Fields] OR “carbonized” [All Fields] OR “carbonizing” [All Fields] OR “carbonous” [All Fields] OR “fizzy” [All Fields]) AND (“nanoparticle s” [All Fields] OR “nanoparticles” [MeSH Terms] OR “nanoparticles” [All Fields] OR “nanoparticle” [All Fields]) AND (“thyroid gland” [MeSH Terms] OR (“thyroid” [All Fields] AND “gland” [All Fields]) OR “thyroid gland” [All Fields] OR “thyroid” [All Fields] OR “thyroid usp” [MeSH Terms] OR (“thyroid” [All Fields] AND “usp” [All Fields]) OR “thyroid usp” [All Fields] OR “thyroids” [All Fields] OR “thyroid s” [All Fields] OR “thyroidal” [All Fields] OR “thyroideal” [All Fields] OR “thyroidism” [All Fields] OR “thyroiditis” [MeSH Terms] OR “thyroiditis” [All Fields] OR “thyroiditides” [All Fields]).

Two independent reviewers (N.T.) and (V.B.) performed the first stage of literature search in the above-mentioned databases and evaluated the articles retrieved for their relevance. In cases of disagreement between the two reviewers, a third, independent reviewer (A.A.A.) was involved and, ultimately, either a consensus was reached, or the majority opinion was used for the final decision on the respective articles. Following removal of duplicate records, a first screening process was performed based on titles and abstracts. Afterwards, a second stage of screening process based on full-text analysis was performed to decide which studies were to be included in the final qualitative and quantitative analysis. Studies that were ultimately included in this meta-analysis had to meet the following criteria: (1) include patients diagnosed with thyroid malignancy, (2) include only adult patients, (3) patients had to be subjected to thyroidectomy or lobectomy and/or neck lymph node dissection and (4) studies had to be designed to compare the use of carbon nanoparticles with the use of methylene blue (MB) or with blank control and (5) the full text was available in English. Animal studies and studies where data were not completely available for extraction were excluded. Also, in cases of two or more studies reported by the same authors and/or institution, either the most recent or the higher quality study was included in the analysis, to exclude possible duplicate cases.

Data extracted from study included the name of the first author, the year of publication, the study period, the number of the participants and their distribution in experimental and control groups, the age of the participants in each group, their sex, the total number of lymph nodes harvested in each group, the number of positive (metastatic) lymph nodes harvested, as well as the number of accidentally removed parathyroid glands in each group. The extracted data were used to compare the average number of total lymph nodes harvested between the carbon nanoparticle infusion group and the control group, as well as to compare the number of metastatic lymph nodes harvested between these groups in order to assess the efficiency of carbon nanoparticle infusion in detecting lymph nodes and especially pathologic ones. The number of accidentally removed parathyroid glands was also compared between the two groups in order to assess the level of parathyroid gland protection that can be achieved by carbon nanoparticle infusion.

All statistical analyses in this meta-analysis were carried out using Reviewer Manager 5.4.1 software (Review Manager (RevMan) [Computer Program]. Version 5.4.1, Copenhagen: The Nordic Cochrane Centre, Denmark, The Cochrane Collaboration, 2020). Data in this study are presented as mean ± standard deviation, as well as weighted mean differences (WMDs) and Odds ratios (ORs) with a confidence interval (CI) of 95%. The assumption of data homogeneity is proven with the use of the chi-squared test and the calculation of the I^2^ index and in cases of homogeneous data a fixed model was used for the statistical analysis, whereas in heterogeneous data a random model was applied. In the current systematic review and meta-analysis, the value of *p* < 0.05 is used as the level of statistical significance, unless stated differently. Potential publication bias was assessed by using Begg’s funnel plot and calculating the respective Egger’s test. Trial Sequential Analysis (TSA) was performed to assess if the study sample size was sufficient to withdraw conclusions or if more studies were required. The software that was used for TSA was Trial Sequential Analysis (TSA) [Computer program]. Version 0.9.5.10 Beta. The Copenhagen Trial Unit, Centre for Clinical Intervention Research, The Capital Region, Copenhagen University Hospital—Rigshospitalet, 2021. Meta-regression analysis was also performed in cases of large heterogeneity.

This systematic review and meta-analysis was prepared according to the PRISMA checklist.

## 3. Results

**Figure 1:**
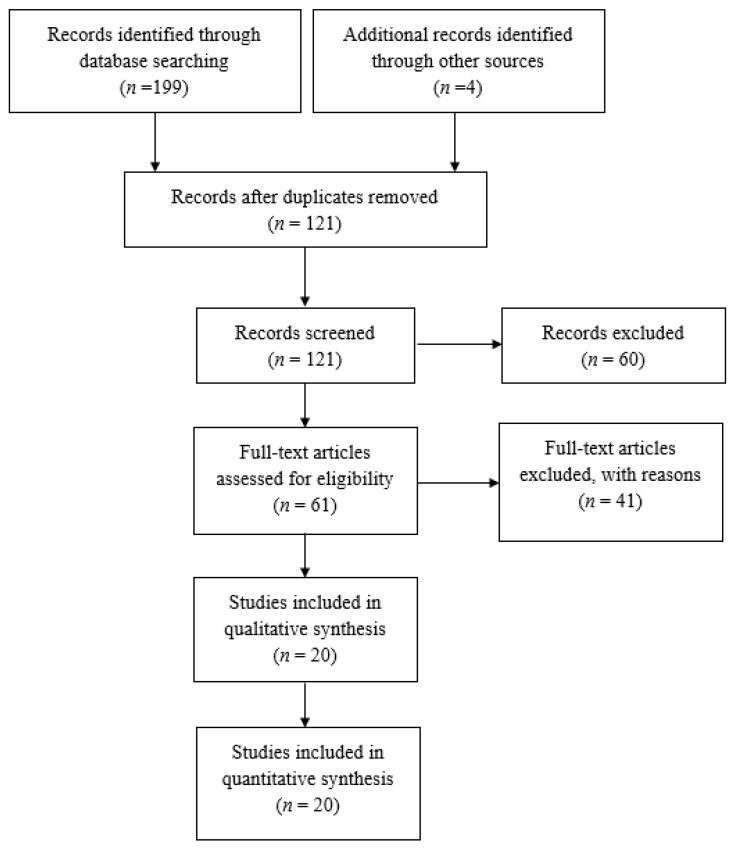
Flowchart depicting the selection process for inclusion of studies in the article.

The original search of the online databases yielded a total number of 199 articles, while 4 more articles were identified by searching the available gray literature, resulting in a total of 203 articles. After excluding duplicate records, the total number of articles was reduced to 121. These articles were initially screened according to their title and abstract and the number was further reduced to 61 articles that were eligible for full-text analysis. After full-text analysis was performed, 41 studies were excluded, as the design, protocol and/or methodology used investigated a different PICO question to those of our study. Ultimately, 20 studies [19,20,24,25,26,27,28,29,30,31,32,33,34,35,36,37,38,39,40,41] were included in the qualitative and quantitative synthesis. The flowchart of the studies selection process is shown in Figure 1.

The publication date of the studies that were included in this systematic review and meta-analysis ranged from 2012 to 2021 with four studies published in 2021 [20,24,25,26]. The shortest study period among the studies was eleven months [35], while the longest one was sixty months [29]. The total number of patients across the 20 studies included in this systematic review and meta-analysis was 2920. Out of the above-mentioned number of patients, 1601 patients in total received carbon nanoparticle suspension during their operation, while the rest 1319 were allocated to control groups. The characteristics of each individual study are shown in Table 1.

Among the studies included, fifteen mentioned the average number of lymph nodes harvested in the experimental and control groups and ten mentioned the number of pathologic (metastatic) lymph nodes harvested compared to the overall number of lymph nodes in both groups. Thirteen studies in total mentioned the number of parathyroid glands that were accidentally removed in the two groups as a measurement of the level of parathyroid gland protection that the use of carbon nanoparticles suspension offers. These data are presented in Table 2.

**Figure 2:**
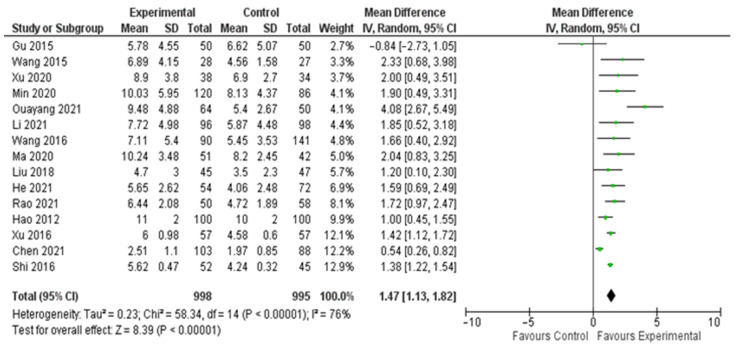
Forest plot of the total number of lymph nodes harvested.

**Figure 3:**
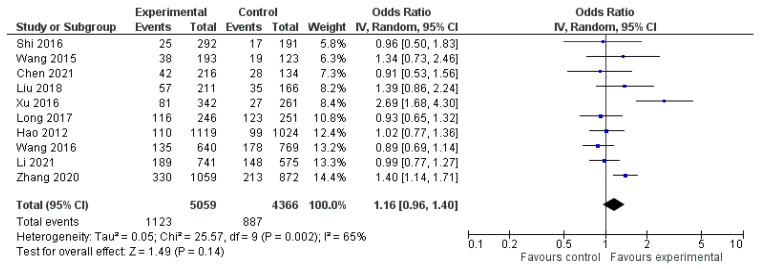
Forest plot of the metastatic lymph nodes harvested.

The meta-analysis on the data from the fifteen studies that mentioned the average number of lymph nodes harvested in the experimental and control groups showed a statistically significant difference in favor of the experimental (carbon nanoparticle suspension) group. More specifically, the WMD was 1.47 (95% CI, 1.13 to 1.82, *p* < 0.001). For this calculation, a random effect model was applied, as data were heterogeneous (I^2^ = 76%, *p* < 0.001). This outcome is presented in Figure 2. Regarding the number of metastatic lymph nodes harvested based on the data retrieved from ten studies, there was no statistically significant difference between the control and the experimental group (OR 1.16, CI 95% 0.96 to 1.40, *p* = 0.14). This calculation was also performed by applying a random effect model as data were again heterogeneous (I^2^ = 65%, *p* = 0.002). The outcome of this analysis is shown in Figure 3.

**Figure 4:**
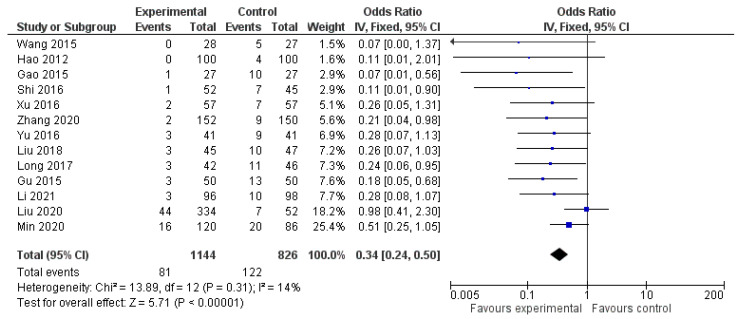
Forest plot of accidentally removed parathyroid glands.

Regarding the number of parathyroid glands that were accidentally removed during the operations, the meta-analysis from the available data from thirteen studies showed that there was a statistically significant 34% decreased chance of accidental removal of a parathyroid gland in the carbon nanoparticle suspension group (OR 0.34, CI 95% 0.24 to 0.50, *p* < 0.001). For this calculation, a fixed model was applied as the data were homogeneous (I^2^= 14%, *p* = 0.31). This outcome is portrayed in Figure 4.

**Figure 5:**
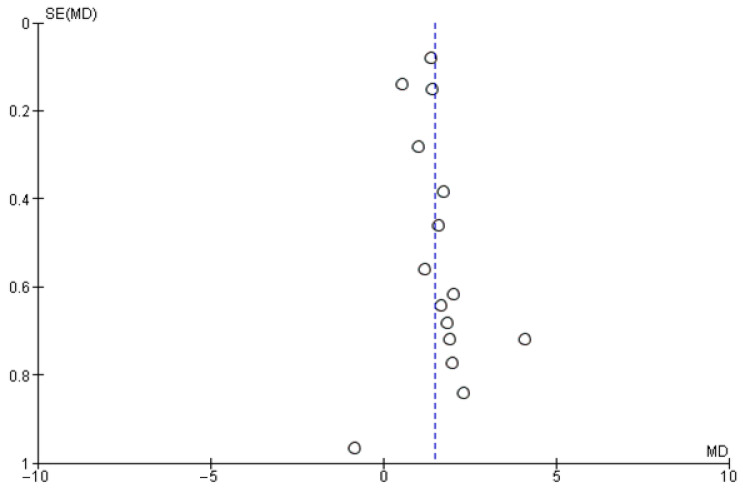
Funnel plot of the studies included in the analysis of the total number of harvested lymph nodes.

**Figure 6:**
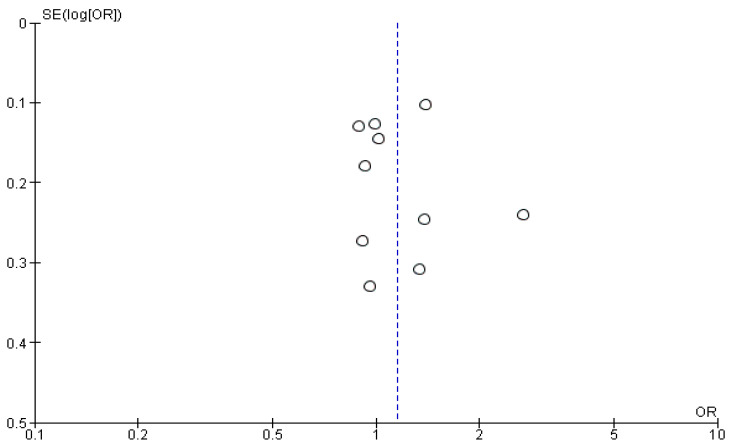
Funnel plot of the studies included in the analysis of the metastatic lymph nodes harvested.

**Figure 7:**
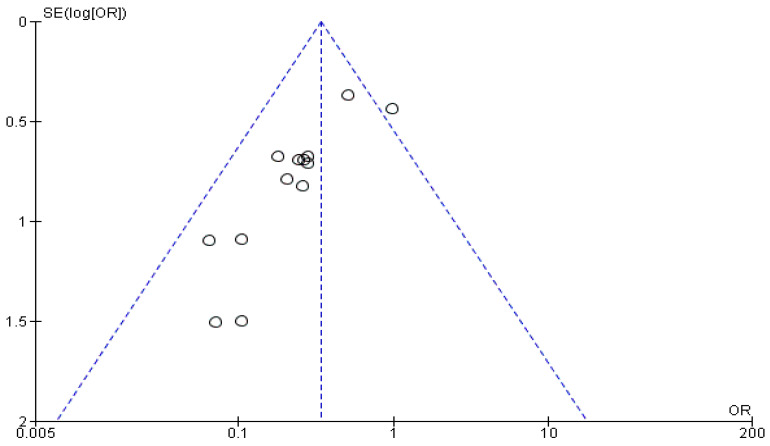
Funnel plot of the studies included in the analysis of the number of accidentally removed parathyroid glands.

The Funnel plots for the studies included in each of the above-mentioned analyses are shown in Figure 5, Figure 6 and Figure 7. In Figure 5, the calculated Egger’s test yielded a value of z = 4.3644 (*p* < 0.0001). In Figure 6, the respective test value was z = −4.3435 (*p* < 0.0001) and in Figure 7 it was z = −2.1671 (*p* = 0.0302). Based on the graphical presentation and the outcome of the Egger’s test, publication bias in our study is unlikely. Meta-regression analysis for the two first analyses where a large heterogeneity was initially identified resulted in an I^2^ value of 38.4% (*p* = 0.0416) and 18.26% (*p* = 0.2098), indicating significant heterogeneity only in the first analysis.

**Figure 8:**
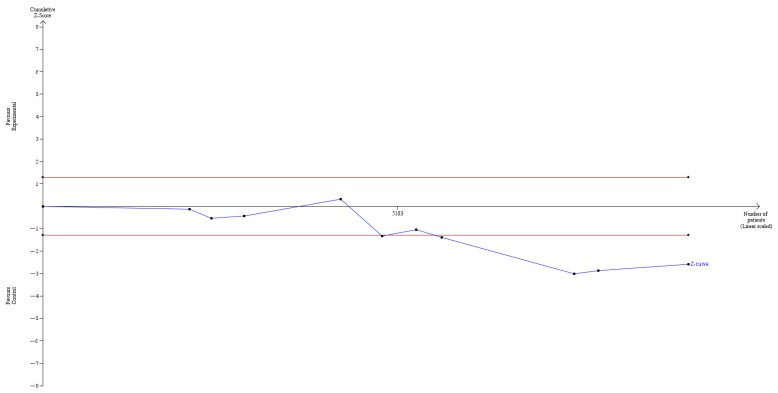
TSA for the number of lymph nodes harvested-z-curve ends outside the not statistically significant zone.

**Figure 9:**
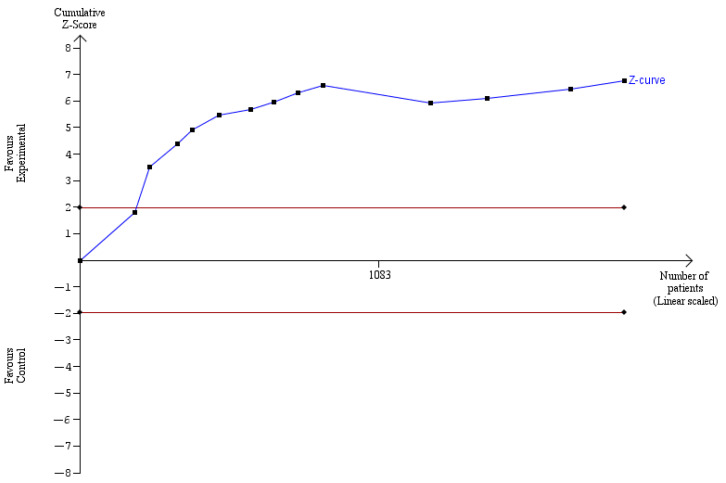
TSA for the number of parathyroid glands removed-z-curve ends outside the not statistically significant zone.

The outcomes of the TSAs performed for the number of metastatic lymph nodes harvested and the number of parathyroid glands that were accidentally removed are portrayed in Figure 8 and Figure 9, respectively, and show that the sample number is sufficient to draw conclusions, and that no further studies are required.

## 4. Discussion

Surgical management is the dominant treatment option in cases of thyroid malignancies, especially for differentiated thyroid carcinomas [42]. More specifically, for papillary thyroid carcinoma the fundamental treatment consists of tumor resection and dissection of the regional lymph nodes [43]. This is mainly because papillary thyroid cancer and its follicular variant spread to the cervical lymph nodes in 20–50% of the cases, with the central neck compartment being predominantly affected, followed by the lateral compartments, although rare cases of skip metastases to the lateral compartments without involvement of the central compartment have been documented [44]. Nonetheless, in certain cases a re-operation will be inevitable, either due to inadequate original treatment or recurrence of the primary disease [45]. In these cases of re-operation, patients usually present with altered anatomy and fibrotic and scarred tissues that increase significantly the complication level of the surgical maneuvers [46].

Thyroid surgery is also associated with several significant complications. Hypocalcemia and hypoparathyroidism are among the most common of these complications, with temporary (transient) hypoparathyroidism ranging from 1.6% to 53.6%, while the incidence of permanent hypoparathyroidism (hypoparathyroidism that persists after six months postoperatively) is 4.11% [47]. This is mainly attributed to the fact that the anatomical location of the parathyroid glands, and especially the lower two, is variable and can lead to accidental removal during thyroid surgery [48]. However, intraoperative identification and exposure of the parathyroid glands facilitates their preservation. As a consequence, novel techniques are required in order to promote the identification and more efficient dissection of neck lymph nodes, as well as the protection of the parathyroid glands during thyroid surgery and especially in cases of reoperation.

Carbon nanoparticles, which have been approved by China Food and Drug Administration, have been recently utilized in thyroid surgery to facilitate lymph node dissection and parathyroid gland identification, as they stain the thyroid gland and its associated lymph nodes black, while the parathyroid glands retain their normal color [23]. The carbon nanoparticle suspension injection consists of active nano-carbon combined with polyvinylpyrrolidone and physiological saline [49] and so far its administration to humans has not been associated with any toxic side effects [2].

In this systematic review and meta-analysis, a total number of 20 studies on the utilization of carbon nanoparticles in thyroid cancer surgery were included. Fifteen of these studies provided data on the average number of lymph nodes harvested in the experimental and control groups and the statistical analysis demonstrated that the use of carbon nanoparticles yields an average number of 1.47 more lymph nodes when compared to the control group. However, there was no statistically significant difference in the ratio of the pathologic (metastatic) lymph nodes harvested between the experimental and control groups, as indicated by the analysis of the data that originated from ten of the studies included in the quantitative analysis. This result indicates that carbon nanoparticles can play a significant role in the tracing of lymph nodes, but they cannot facilitate a better identification of metastatic lymph nodes. This can most likely be attributed to the fact that carbon nanoparticles do not have tumor tropism and as a result they stain both normal and pathologic lymph nodes at the same rate [32]. Moreover, regarding parathyroid gland protection, our meta-analysis showed that the use of carbon nanoparticles reduces the chances of accidental removal of a parathyroid gland by 34%, indicating that their use can significantly reduce the rate of post-operative hypoparathyroidism. These findings corroborate previous studies on the subject [2].

In addition, other dyes have been used in order to improve the outcomes of thyroid surgery, such as methylene blue and indocyanine green [50,51]. However, the main disadvantage of methylene blue is that not only does it stain the lymph nodes, but the parathyroid glands as well, which leads to intraoperative problems [52]. More specifically, in a study performed in 2012 by Hao et al. [33], the use of carbon nanoparticles was superior to methylene blue in terms of lymph node staining and positivity rate for metastatic disease of the sentinel lymph node. In addition, the use of methylene blue has been associated with heart abnormalities as well as neurotoxic effects in patients who receive serotonergic medication [53,54]. Also, based on two studies performed by Zhang et al. in 2019 and 2020 [18,55] the use of indocyanine green alone is superior to carbon nanoparticles alone in identifying lymph nodes, but the combined technique can result in more metastatic lymph nodes harvested.

The current systematic review and meta-analysis has specific limitations when it comes to interpretation of its results. Most importantly, all of the included studies originate from China and therefore all the patients included were Chinese. It is still unclear whether similar outcomes can be expected by the application of this technique in other countries. Moreover, this meta-analysis did not focus on the long-term outcomes of the use of carbon nanoparticles regarding the recurrence rate and prognosis of thyroid malignancies or the long-term recovery of parathyroid function. Therefore, further research will be needed to consolidate the findings of this study.

## 5. Conclusions

This systematic review and meta-analysis aimed to evaluate the use of carbon nanoparticles in thyroid cancer surgery. It demonstrated that the administration of carbon nanoparticle suspension during primary surgery or re-operation can increase the number of lymph nodes detected and harvested as well as improve parathyroid gland protection, as it leads to a significant decrease in accidental intraoperative removal.

## Figures and Tables

**Table 1:** Characteristics of each individual study included in the meta-analysis (all studies originate from China).

Study	Study Period	Experimental Group (*n*, Age)	Control Group (*n*, Age)	Sex (Male/Female)	Study Design
Liu, et al. [19]	November 2017 to October 2018	334, 44.0 ± 11.7	52, 46.6 ± 13.0	98/288	Retrospective
He, et al. [20]	January 2018 to December 2020	54, 34.7 ± 9.8	72, 35.0 ± 9.1	4/122	Retrospective
Rao, et al. [24]	January 2015 to April 2019	50, 46.8 ± 11.9	58, 44.0 ± 10.2	25/83	Randomized, control trial
Chen, et al. [25]	September 2019 to December 2020	103, 48.57 ± 13.01	88, 45.49 ± 13.25	46/145	Randomized, control trial
Ouyang, et al. [26]	March 2020 to March 2021	64, 34.72 ± 8.79	50, 37.24 ± 9.63	17/97	Retrospective
Li, et al. [27]	February 2017 to April 2019	96, 42.54 ± 12.49	98, 43.86 ± 12.35	37/157	Retrospective
Min, et al. [28]	2017 to 2018	120, 40.05 ± 12.37	86, 39.63 ± 11.22	44/162	Retrospective
Ma, et al. [29]	June 2014 to June 2019	51, 31.8 ± 7.3	42, 30.2 ± 9.2	13/80	Randomized, control trial
Xu, et al. [30]	January 2017 to January 2019	38, 30.5 ± 7.0	34, 32.6 ± 7.2	6/66	Retrospective
Zhang, et al. [31]	February 2016 to June 2018	152, 33.5 ± 10.02	150, 34.1 ± 10.13	22/280	Randomized, control trial
Liu, et al. [32]	February 2013 to May 2015	45, 46.17 ± 10.20	47, 45.39 ± 12.03	29/63	Prospective
Xu, et al. [33]	September 2013 to August 2014	57, 45.37 ± 10.71	57, 42.68 ± 14.43	9/105	Randomized control trial
Long, et al. [34]	January 2012 to May 2013	49, 44.5 ± 9.6	54, 43.8 ± 10.3	20/68	Randomized, control trial
Yu, et al. [35]	August 2012 to June 2013	41, 41.6 ± 17.1	41, 41.7 ± 18.9	19/63	Randomized, control trial
Shi, et al. [36]	January 2014 to February 2015	52, 45.2 ± 5.8	45, 42 ± 4.3	12/85	Not mentioned
Wang, et al. [37]	January 2013 to January 2014	90, 44.36 ± 11.48	141, 44.09 ± 12.41	62/169	Prospective
Wang, et al. [38]	March 2013 to March 2014	28, 30.25 ± 6.04	27, 29.44 ± 6.27	3/52	Randomized, control trial
Gu, et al. [39]	June 2012 and August 2014	50, 46.98 ± 9.027	50, 46.98 ± 9.027	16/84	Randomized, control trial
Gao, et al. [40]	January 2012 to December 2014	27, 49.4 ± 2.5	27, 52.5 ± 1.8	4/50	Randomized control trial
Hao, et al. [41]	January 2008 to December 2009	100, 41	100, 44	25/175	Retrospective

**Table 2:** Primary outcomes of each study included in the meta-analysis.

Study	Lymph Nodes Harvested (Experimental Group)	Lymph Nodes Harvested (Control Group)	Metastatic/Total Lymph Nodes Harvested (Experimental Group)	Metastatic/Total Lymph Nodes Harvested (Control Group)	Parathyroid Glands Removed (Experimental Group)	Parathyroid Glands Removed (Control Group)
Liu, et al. [19]	Not mentioned	Not mentioned	Not mentioned	Not mentioned	44/334	7/52
He, et al. [20]	5.65 ± 2.62	4.06 ± 2.48	Not mentioned	Not mentioned	Not mentioned	Not mentioned
Rao, et al. [24]	6.44 ± 2.08	4.72 ± 1.89	Not mentioned	Not mentioned	Not mentioned	Not mentioned
Chen, et al. [25]	2.51 ± 1.10	1.97 ± 0.85	42/216	28/134	Not mentioned	Not mentioned
Ouyang, et al. [26]	9.48 ± 4.88	5.40 ± 2.67	Not mentioned	Not mentioned	Not mentioned	Not mentioned
Li, et al. [27]	7.72 ± 4.98	5.87 ± 4.48	189/741	148/575	3/96	10/98
Min, et al. [28]	10.03 ± 5.95	8.13 ± 4.37	Not mentioned	Not mentioned	16/120	20/86
Ma, et al. [29]	10.24 ± 3.48	8.20 ± 2.45	Not mentioned	Not mentioned	Not mentioned	Not mentioned
Xu, et al. [30]	8.9 ± 3.8	6.9 ± 2.7	Not mentioned	Not mentioned	Not mentioned	Not mentioned
Zhang, et al. [31]	Not mentioned	Not mentioned	330/1059	213/872	2/152	9/150
Liu, et al. [32]	4.7 ± 3.0	3.5 ± 2.3	57/211	35/166	3/45	10/47
Xu, et al. [33]	6.00 ± 0.98	4.58 ± 0.60	81/342	27/261	2/57	7/57
Long, et al. [34]	Not mentioned	Not mentioned	116/246	123/251	3/42	11/46
Yu, et al. [35]	Not mentioned	Not mentioned	Not mentioned	Not mentioned	3/41	9/41
Shi, et al. [36]	5.62 ± 0.47	4.24 ± 0.32	25/292	17/191	1/52	7/45
Wang, et al. [37]	7.11 ± 5.4	5.45 ± 3.53	135/640	178/769	Not mentioned	Not mentioned
Wang, et al. [38]	6.89 ± 4.15	4.56 ± 1.58	38/193	19/123	0/28	5/27
Gu, et al. [39]	5.78 ± 4.55	6.62 ± 5.07	Not mentioned	Not mentioned	3/50	13/50
Gao, et al. [40]	Not mentioned	Not mentioned	Not mentioned	Not mentioned	1/27	10/27
Hao, et al. [41]	11 ± 2	10 ± 2	110/1119	99/1024	0/100	4/100
